# Supplementary material for: Synthesis, Biological Activity and Molecular Docking Studies of Novel Nicotinic Acid Derivatives
Source: Int J Mol Sci. 2022 Mar 4;23(5):2823. doi: 10.3390/ijms23052823 (PMC8911400; doi:10.3390/ijms23052823)
Supplement: Supplementary file 1 [file ijms-23-02823-s001.zip › ijms-1605869-supplementary.pdf]

Synthesis, Biological Activity and Molecular Docking Studies of Novel Nicotinic Acid Derivatives

Kinga Paruch [1]*, Anna Biernasiuk [2], Dmytro Khylyuk [1], Roman Paduch [3], Monika Wujec [1] and Łukasz Popiołek [1]

[1] Chair and Department of Organic Chemistry, Faculty of Pharmacy, Medical University of Lublin, 4A Chodźki Street, 20-093 Lublin, Poland; dmytro.khylyuk@umlub.pl (D.K.); monika.wujec@umlub.pl (M.W.); lukasz.popiolek@umlub.pl (Ł.P.);
[2] Chair and Department of Pharmaceutical Microbiology, Faculty of Pharmacy, Medical University of Lublin, 1 Chodźki Street, 20-093 Lublin, Poland; anna.biernasiuk@umlub.pl (A.B.);
[3] Department of Virology and Immunology, Institute of Biological Sciences, Faculty of Biology and Biotechnology, Maria Curie-Skłodowska University, 19 Akademicka Street, 20-033 Lublin, Poland; rpaduch@poczta.umcs.lublin.pl (R.P.);
* Correspondence: kinga.paruch@umlub.pl

SUPPLEMENTARY MATERIALS

Materials and methods

Chemistry

Representative ^{1}H NMR and ^{13}C NMR spectra of acylhydrazones of nicotinic acid (**2**–**13**):

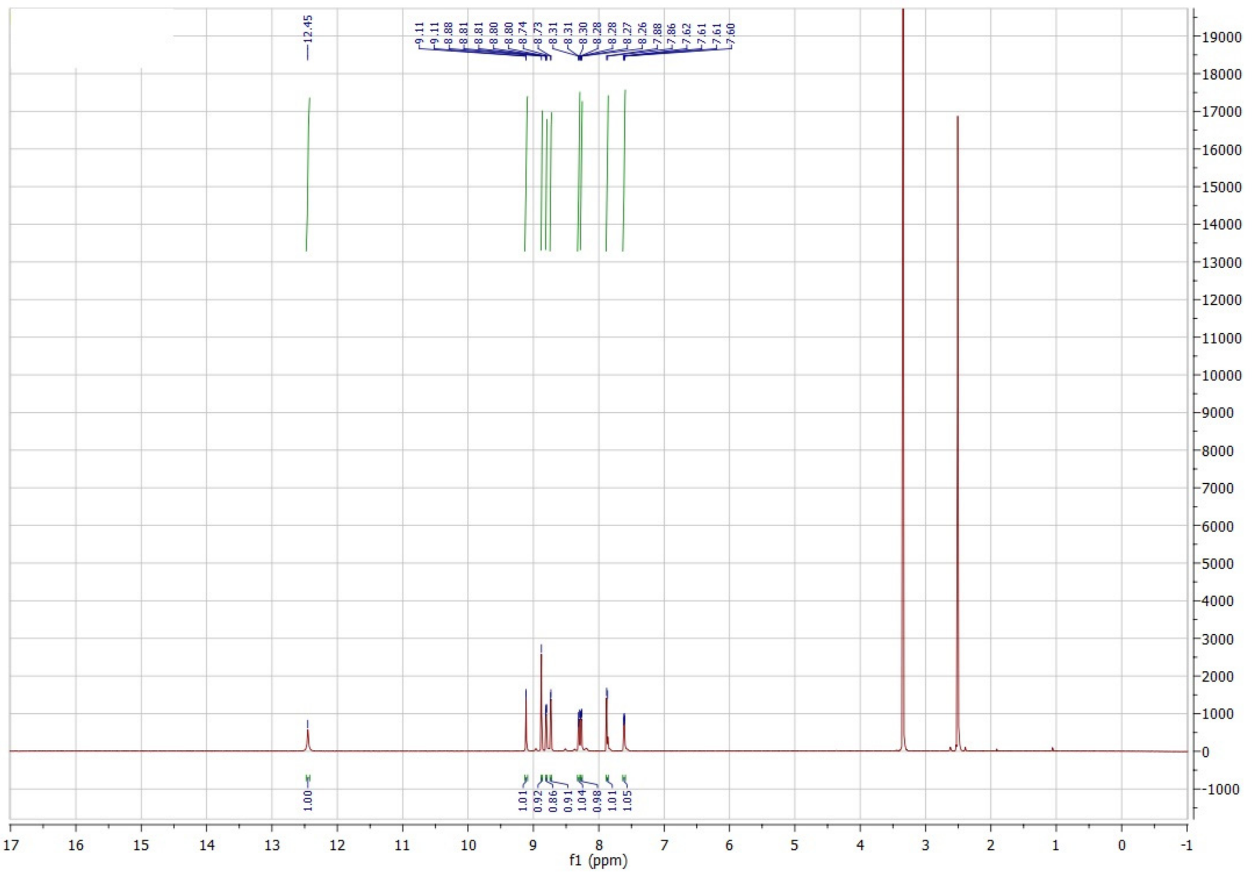

Figure S1. The ^{1}H NMR spectra of compound **10.**

Compound **10**: *N*-[(2-chloro-5-nitrophenyl)methylidene]pyridine-3-carbohydrazide

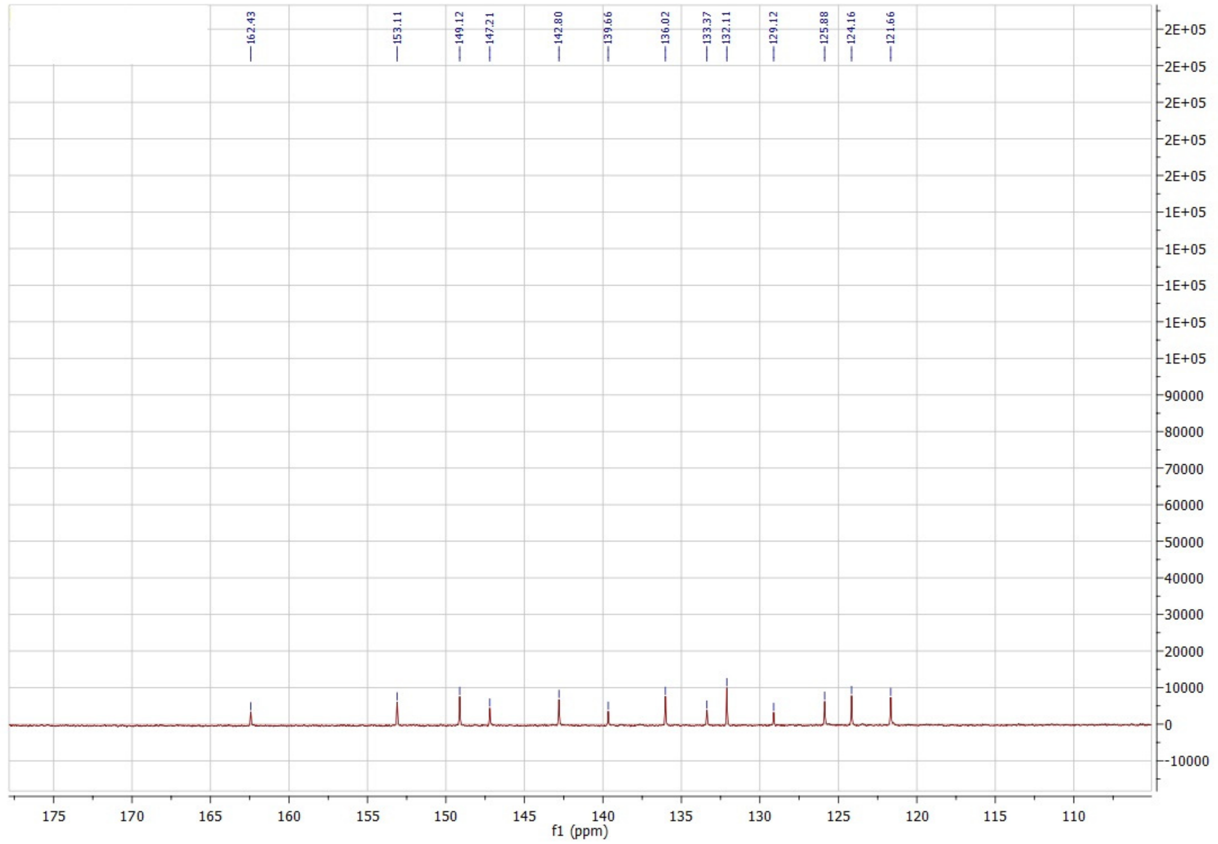

Figure S2. The ^{13}C NMR spectra of compound **10.**

Compound **10**: *N*-[(2-chloro-5-nitrophenyl)methylidene]pyridine-3-carbohydrazide

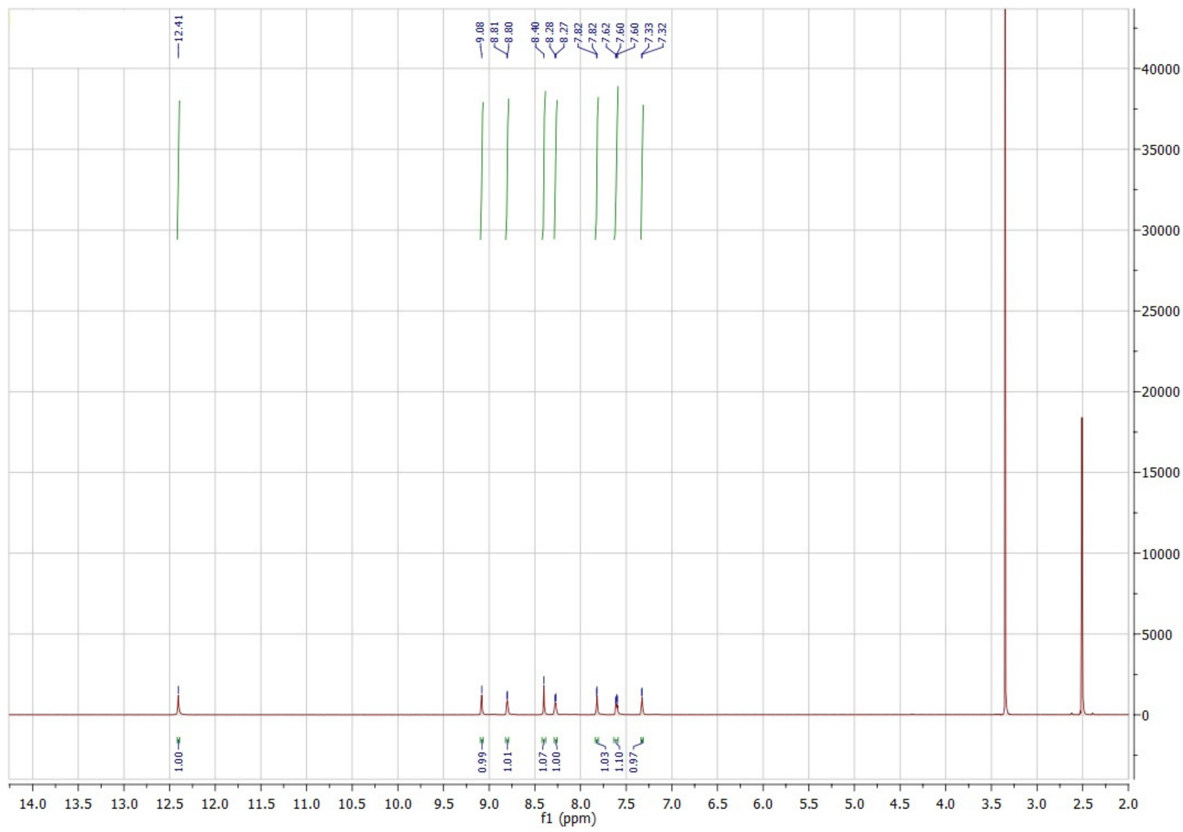

Figure S3. The ^{1}H NMR spectra of compound **13.**

Compound **13**: *N*-[(5-nitrofuran-2-yl)methylidene]pyridine-3-carbohydrazide

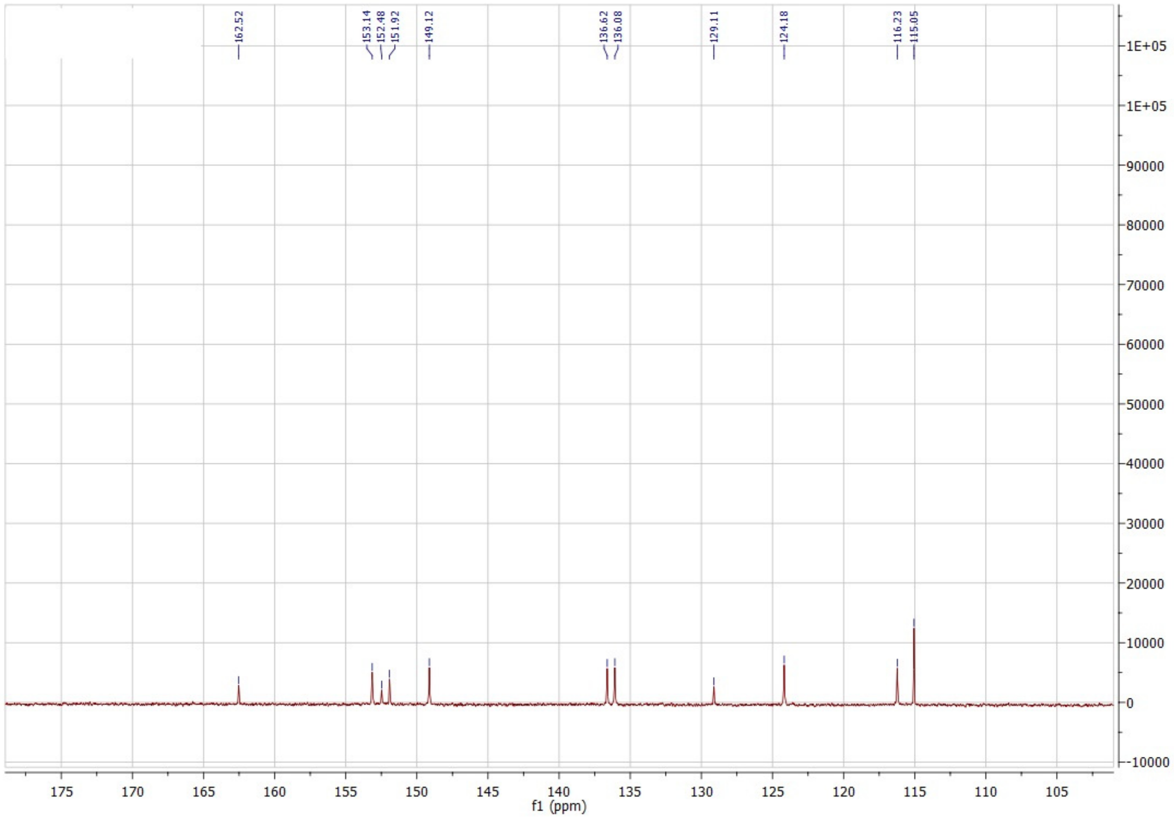

Figure S4. ^{13}C NMR spectra of compound **13.**

Compound **13**: *N*-[(5-nitrofuran-2-yl)methylidene]pyridine-3-carbohydrazide

Representative ^{1}H NMR and ^{13}C NMR spectra of 3-acetyl-2,5-disubstituted-1,3,4-oxadiazoline derivatives (**14-25**):

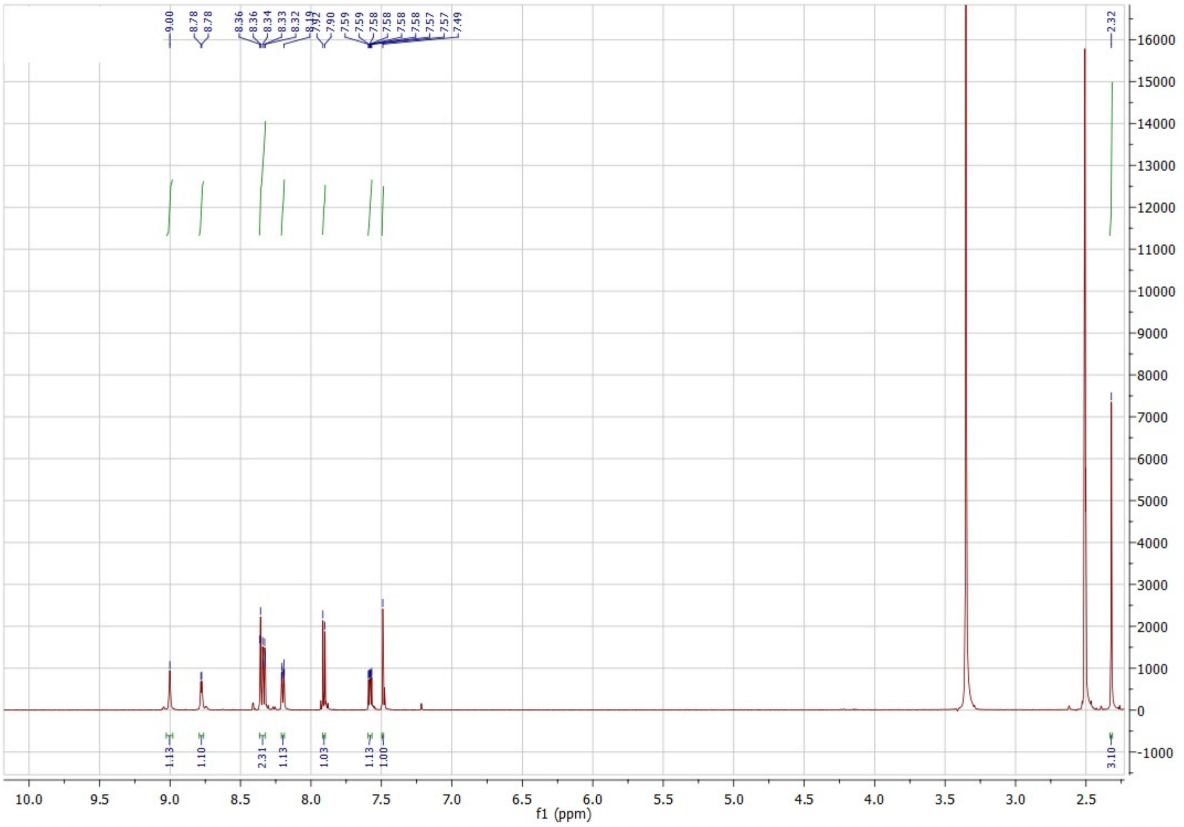

Figure S5. ^{1}H NMR spectra of compound **22.**

Compound **22**: 1-[2-(2-chloro-5-nitrophenyl)-5-(pyridin-3-yl)-1,3,4-oxadiazol-3(2*H*)-yl]ethan-1-one

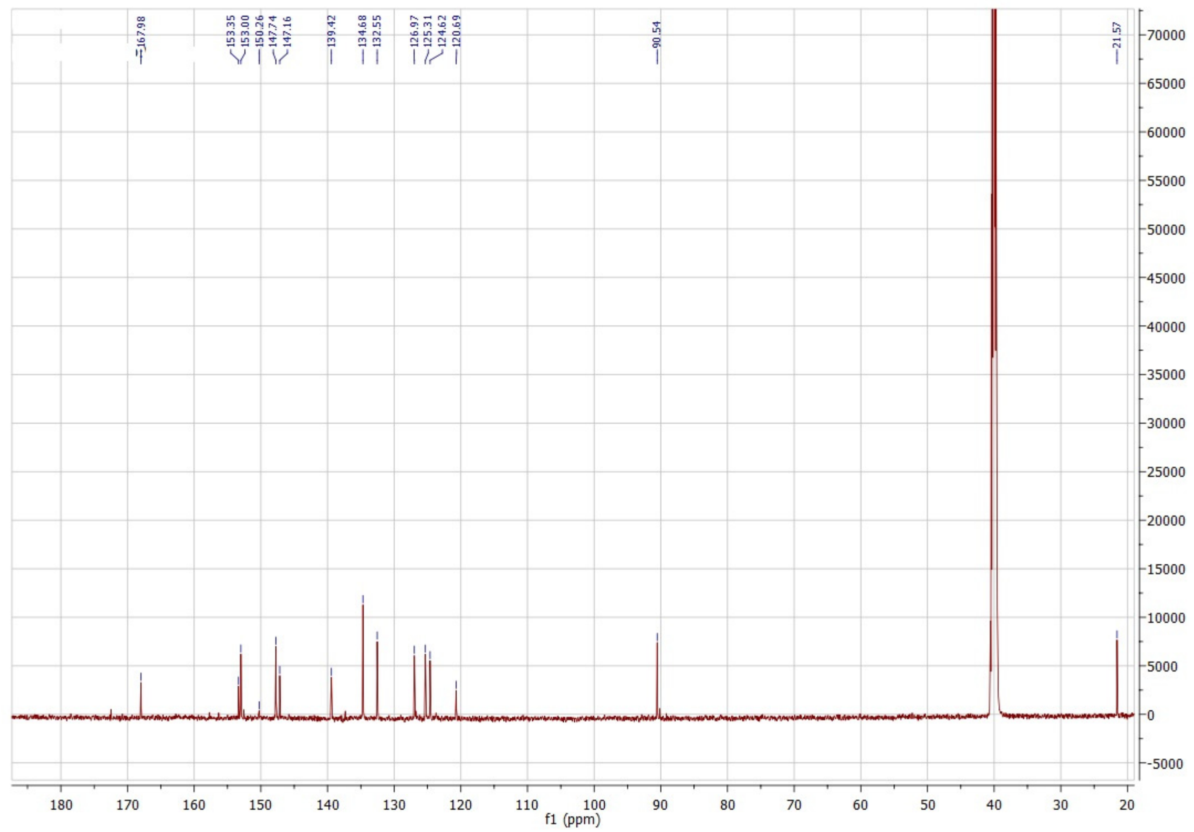

Figure S6. ^{13}C NMR spectra of compound **22.**

Compound **22**: 1-[2-(2-chloro-5-nitrophenyl)-5-(pyridin-3-yl)-1,3,4-oxadiazol-3(2*H*)-yl]ethan-1-one

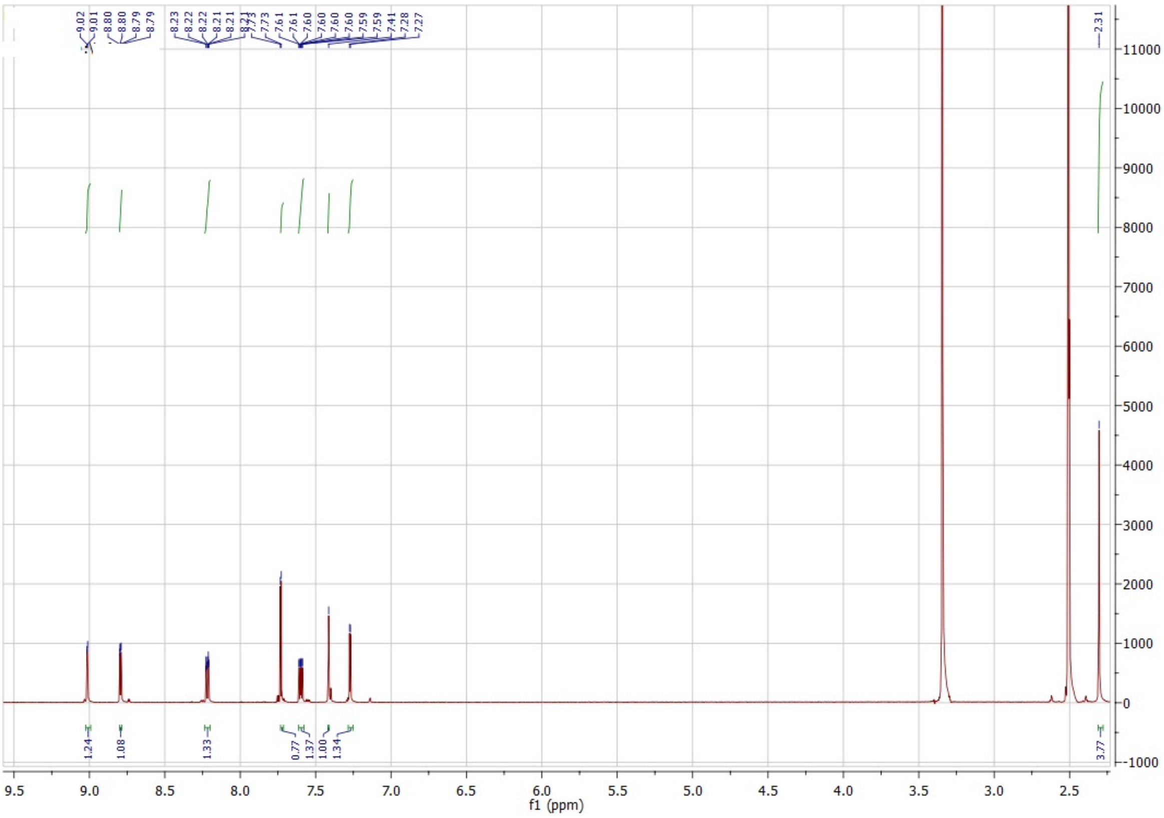

Figure S7. ^{1}H NMR spectra of compound **25.**

Compound **25**: 1-[2-(5-nitrofuran-2-yl)-5-(pyridin-3-yl)-1,3,4-oxadiazol-3(2*H*)-yl]ethan-1-one

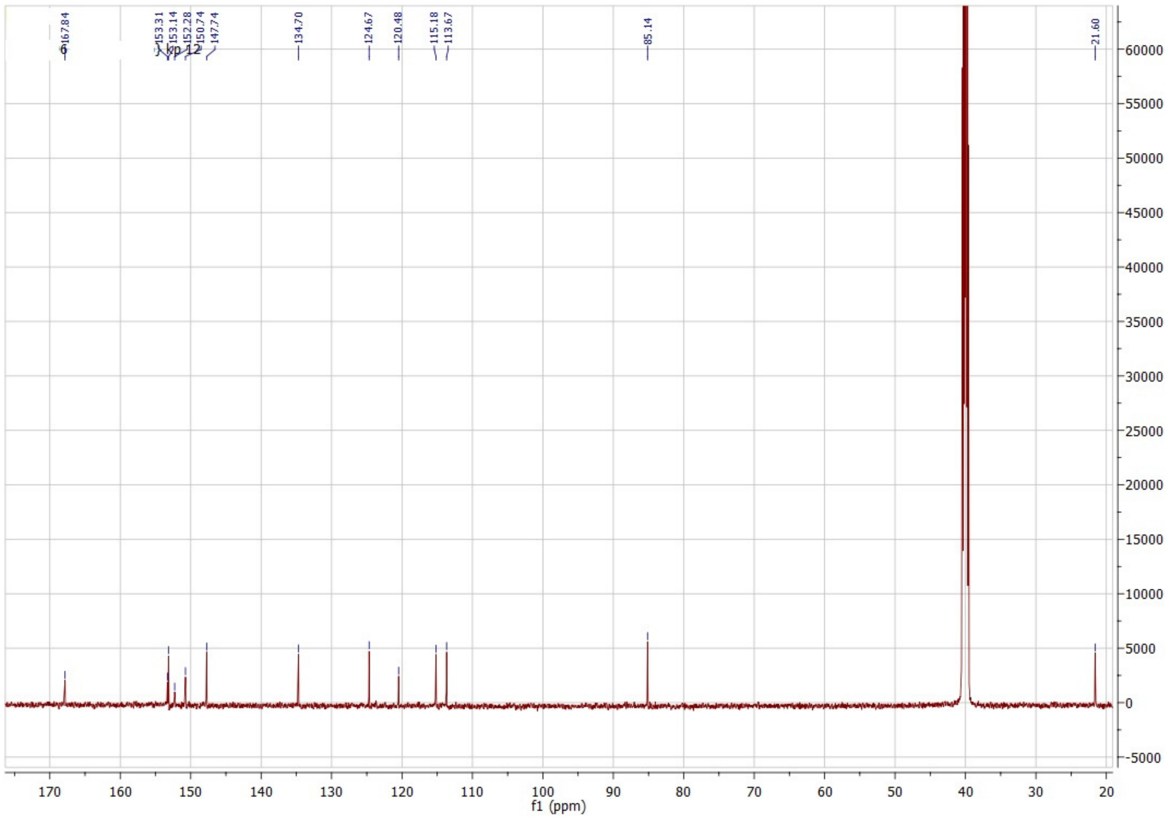

Figure S8. ^{13}C NMR spectra of compound **25.**

Compound **25**: 1-[2-(5-nitrofuran-2-yl)-5-(pyridin-3-yl)-1,3,4-oxadiazol-3(2*H*)-yl]ethan-1-one

May-Grünwald staining after HT29 and CCD 841 CoTr cells incubation with compound **17**

HT29 cells

Control

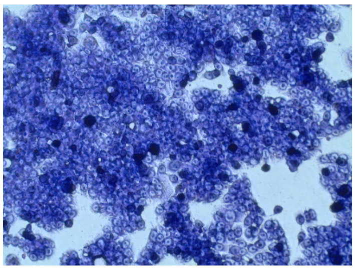

25 µg/ml

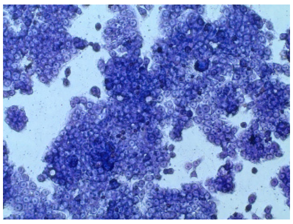

75 µg/ml

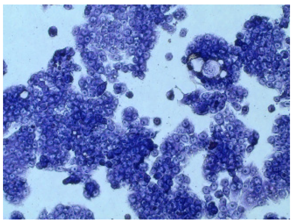

150 µg/ml

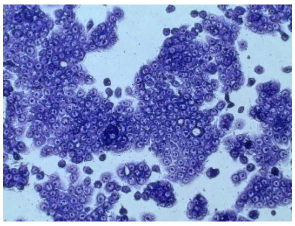

200 µg/ml

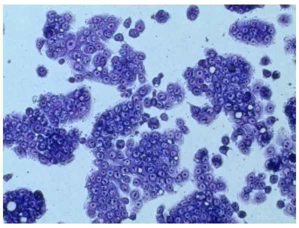

CCD 841 CoTr cells

Control

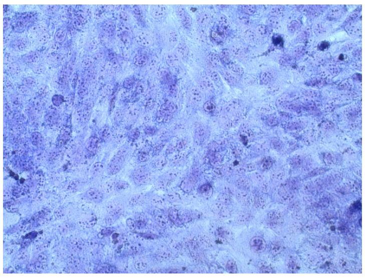

25 µg/ml

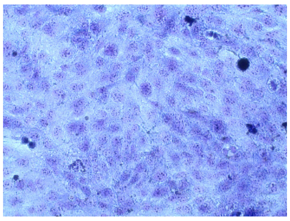

75 µg/ml

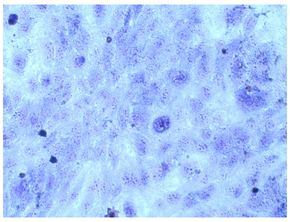

150 µg/ml

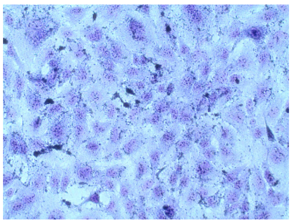

200 µg/ml

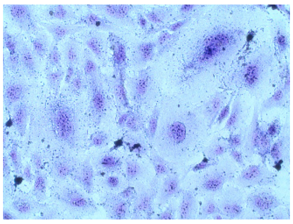

Figure S9. May-Grünwald-Giemsa (MGG) staining results for the compounds **17**, **20**, **21**, **22.**

May-Grünwald staining after HT29 and CCD 841 CoTr cells incubation with compound **20**

HT29 cells

Control

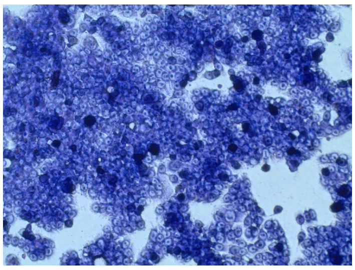

25 μg/ml **75 μg/ml** **150 μg/ml** **200 μg/ml**

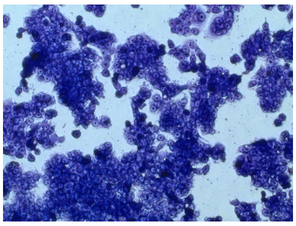
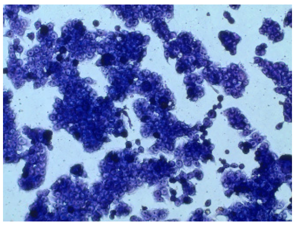
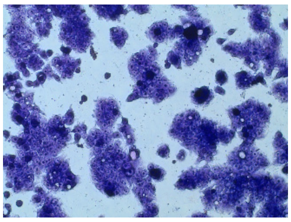
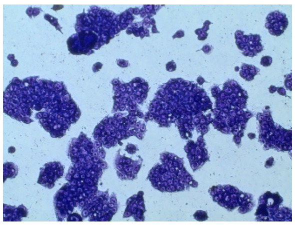

CCD 841 CoTr cells

Control

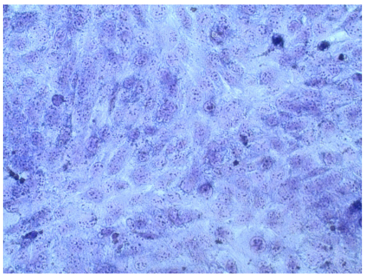

25 μg/ml **75 μg/ml** **150 μg/ml** **200 μg/ml**

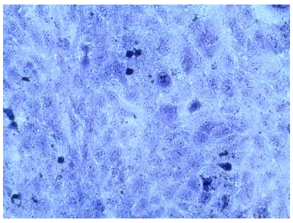
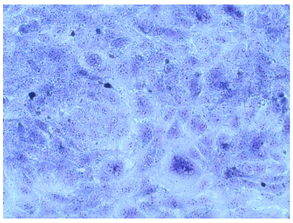
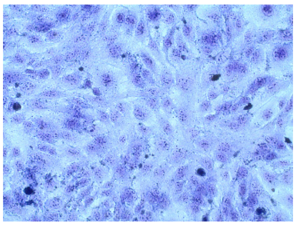
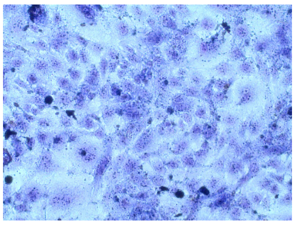

May-Grünwald staining after HT29 and CCD 841 CoTr cells incubation with compound **21**

HT29 cells

Control

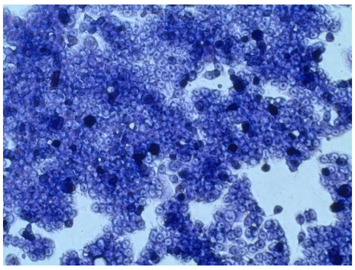

25 μg/ml

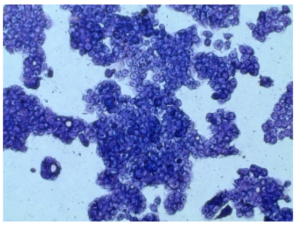

75 μg/ml

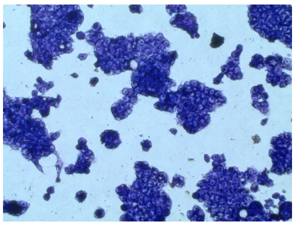

150 μg/ml

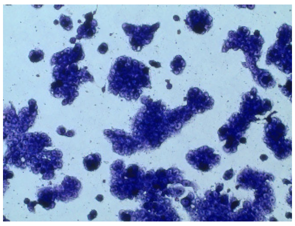

200 μg/ml

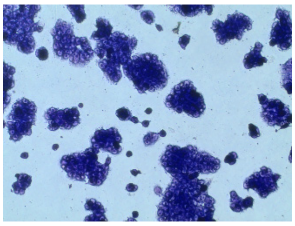

CCD 841 CoTr cells

Control

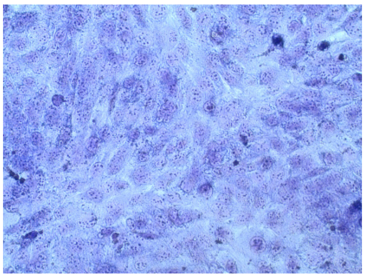

25 μg/ml

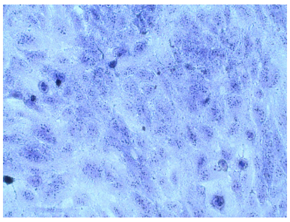

75 μg/ml

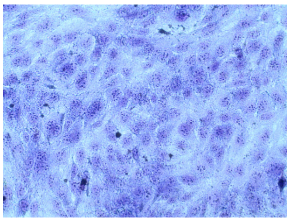

150 μg/ml

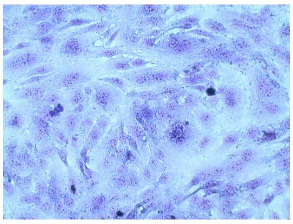

200 μg/ml

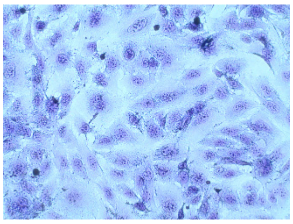

May-Grünwald staining after HT29 and CCD 841 CoTr cells incubation with compound **22**

HT29 cells

Control

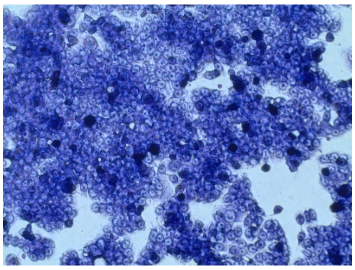

25 μg/ml **75 μg/ml** **150 μg/ml** **200 μg/ml**

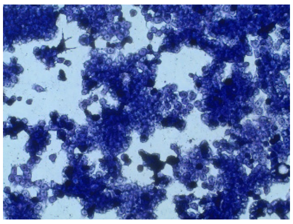
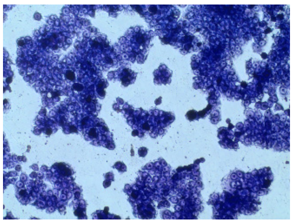
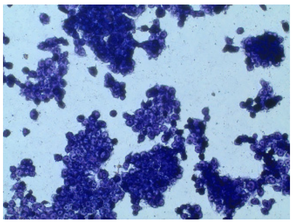
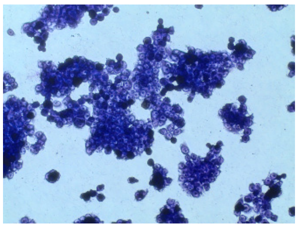

CCD 841 CoTr cells

Control

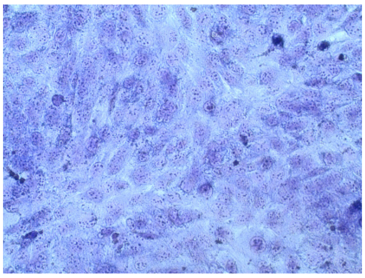

25 μg/ml **75 μg/ml** **150 μg/ml** **200 μg/ml**

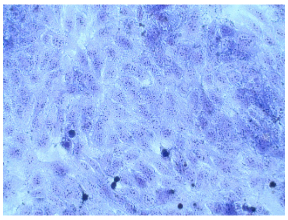
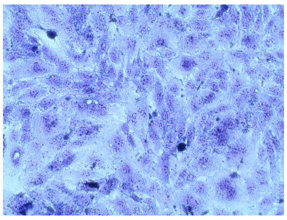
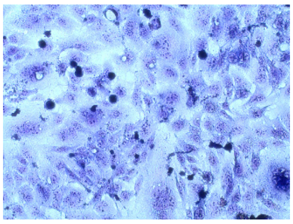
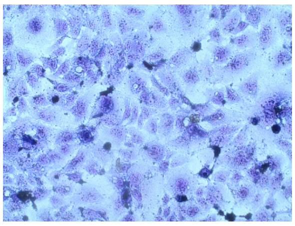

HT29, human colon adenocarcinoma cell line (ATCC No. HTB-38)
CCD 841 CoTr, human normal colon epithelial cells (ATCC No. CRL-1807)
